# ITGB1 alleviates osteoarthritis by inhibiting cartilage inflammation and apoptosis via activating cAMP pathway

**DOI:** 10.1186/s13018-023-04342-y

**Published:** 2023-11-08

**Authors:** Lifeng Xie, Zhengnan Li, Zhijun Chen, Mingzhang Li, Jun Tao

**Affiliations:** 1 Department of Orthopedics, Second Affiliated Hospital of Nanchang University, No.1 MinDe Road, Donghu District, Nanchang City, 330000 Jiangxi Province China; 2 Department of Sports Medicine, The Affiliated Ganzhou Hospital of Nanchang University (Ganzhou People’s Hospital), No.16, MeiGuan Road, Zhanggong District, Ganzhou City, 341000 Jiangxi Province China

**Keywords:** Osteoarthritis, Bioinformatics analysis, ITGB1, Inflammation, Apoptosis, cAMP signaling pathway

## Abstract

**Objective:**

We aimed to screen novel biomarkers for osteoarthritis (OA) using bioinformatic methods and explore its regulatory mechanism in OA development.

**Methods:**

Differentially expressed genes were screened out from GSE98918 and GSE82107 datasets. Protein–protein interaction network and enrichment analysis were employed to search for hub gene and regulatory pathway. Hematoxylin–eosin, Safranin O-Fast green staining, and immunohistochemistry were performed to assess pathological damage. TNF-α, IL-1β, and IL-6 concentrations were determined by enzyme-linked immunosorbent assay. Real-time quantitative PCR was applied to verify expression of hub genes in OA model. The expression of key protein and pathway proteins was determined by western blot. Furthermore, Cell Counting Kit-8 and flow cytometry were conducted to explore the role of hub gene in chondrocytes.

**Results:**

We identified 6 hub genes of OA, including ITGB1, COL5A1, COL1A1, THBS2, LAMA1, and COL12A1, with high prediction value. ITGB1 was screened as a pivotal regulator of OA and cAMP pathway was selected as the key regulatory pathway. ITGB1 was down-regulated in OA model. ITGB1 overexpression attenuated pathological damage and apoptosis in OA rats with the reduced levels of TNF-α, IL-1β and IL-6. ITGB1 overexpression activated cAMP pathway in vivo and vitro models. In vitro model, ITGB1 overexpression promoted cell viability, while inhibited apoptosis. ITGB1 overexpression also caused a decrease of TNF-α, IL-1β, and IL-6 concentrations. cAMP pathway inhibitor reversed the positive effect of ITGB1 on OA cell model.

**Conclusion:**

ITGB1 is a novel biomarker for OA, which inhibits OA development by activating the cAMP pathway.

**Supplementary Information:**

The online version contains supplementary material available at 10.1186/s13018-023-04342-y.

## Introduction

Osteoarthritis (OA), a chronic degenerative disease, is one of the most prevalent types of arthritis, which is the major inducer of disability [1]. The symptoms of OA mainly manifest as pain, transient morning stiffness, and enlarged deformities of the joints, leading to physical instability and disability, thus affecting the life quality of patients, as well as increasing socioeconomic burden to the patients and the society [2–4]. A recent study has reported that age, gender, obesity, and joint injury are potential risk for OA, affecting 16% of the population worldwide [5]. Notably, the prevalence of OA is growing rapidly due to aging and the prevalence of obesity [3, 6, 7]. Current treatment for OA is mainly limited to non-steroidal anti-inflammatory medications, which relieves symptoms of pain and inflammation [8]. Patients with severe OA require joint replacement surgery, which has a negative impact on patients’ daily activities [9]. Therefore, it is essential to explore possible molecular targets for abating the joint inflammatory response.

Inflammatory mediator plays a vital role in OA pathogenesis, causing morphologic alterations within cartilage degradation, osteophyte production, and synovitis [10]. Additionally, inflammatory factors have an important effect on the activation of downstream signaling pathways, thus triggering chondrocyte hypertrophy, dedifferentiation, and ultimately chondrocyte apoptosis [10–13]. Moreover, apoptosis of chondrocytes has a positive correlation with the severity of OA [14, 15]. An increase in apoptotic cells in articular cartilage also has been observed in both early and late stages of OA [13, 15]. The massive release of pro-inflammatory mediators has been evidenced to induce chondrocyte apoptosis and exacerbate cartilage matrix degradation, thereby promoting OA progression [16–18]. Therefore, searching for potential molecular mechanisms that prevent inflammation and apoptosis may provide new options for clinical treatment of OA.

Integrin β1 (ITGB1), a member of the integrin family, involves in receptor-mediated activity, cell–matrix adhesion, cell signaling, cell defense, cell adhesion, protein binding, and protein heterodimerization [19–22]. ITGB1 protein expression levels are down-regulated in bone marrow and adipose tissue-derived stem cells, while ITGB1 expression is significantly up-regulated during chondrogenic differentiation of these bone marrow mesenchymal stem cells, and ITGB1 promotes the differentiation of Adipose-derived mesenchymal stem cells toward cartilage [23, 24]. In addition, ITGB1 has been reported to promote osteoblast differentiation and the formation of osteoblast extracellular matrix [25]. High expression of ITGB1 in the human body has a key effect on extracellular matrix, cytoskeleton, and signaling proteins for maintaining chondrocyte phenotypes, suppressing chondrocyte apoptosis, and regulating chondrocyte specific gene expression [26]. However, the role of ITGB1 in the development of OA needs to be further investigated.

Various physiological functions, including memory and learning, heart muscle relaxation and contraction, inflammation, and immunological function, are mediated by cyclic adenosine monophosphate (cAMP) pathways [27, 28]. Moreover, cAMP also plays a crucial role in calcium homeostasis, gene transcription, glutaminergic receptor trafficking, cell death, and neurotransmission [29, 30]. Studies have reported cAMP dysregulation is associated with cancer, diabetes, kidney disease, and neurological disorders, etc. [31–33]. Recently, cAMP has been reported as a crucial coordinator in inflammation resolution [34]. Nonetheless, the significance of cAMP in OA is still unknown and needs to be further investigated.

In the present research, based on GSE98918 and GSE82107 datasets, we determined the promising hub genes in the pathogenesis of OA. Furthermore, the abnormal expression of hub genes was confirmed by constructing OA rat model. ITGB1 was selected as a novel signature involved in OA development, and cAMP pathway was the potential mechanism of ITGB1 regulating OA. Specifically, we further explored the specific mechanism of ITGB1 intervention on OA progression through cAMP pathway in lipopolysaccharide (LPS)-induced chondrocyte inflammation in vitro. We hope to provide a potential theoretical basis and therapeutic target for alleviating OA, and ITGB1 is expected to become a promising therapeutic strategy.

## Materials and methods

### Data source and differentially expressed genes (DEGs) identification

The gene expression data analyzed in this research were retrieved from the Gene Expression Omnibus (GEO, https://www.ncbi.nih.gov/geo/). An advanced retrieval procedure was performed by searching keyword: “osteoarthritis.” Two datasets (GSE98918 and GSE82107) were determined for the subsequent research.

GSE98918 and GSE82107 datasets were analyzed using GEO2R (www.ncbi.nlm.nih.gov/geo/geo2r). |logFC|≥ 1 and *p* ≤ 0.05 were the cut-off criteria for DEGs selection (OA vs. Normal). The DEGs of the two datasets were subsequently visualized and the overlapped DEGs between GSE98918 and GSE82107 datasets were identified. Venn map was generated using the Venn Diagrams Software for the display of the common DEGs.

### Gene ontology (GO) and Kyoto encyclopedia of genes and genomes (KEGG) enrichment analysis of DEGs

The common DEGs were subjected to function and pathway enrichment analysis by employing the Database for Annotation, Visualization, and Integrated Discovery (DAVID) software (https://david.ncifcrf.gov/summary.jsp). The GO enrichment terms were as follows: cellular component (CC), biological process (BP) and molecular function (MF). *p* < 0.05 was regarded as statistically significant.

### Protein–protein interaction (PPI) network construction and analysis of hub gene

The PPI network of DEGs was constructed using Search Tool for the Retrieval of Interacting Genes (STRING, https://www.string-db.org/) and visualized using Cytoscape software(www.cytoscape.org/). The key modules in PPI network were then identified using Molecular Complex Detection (MCODE) and further analyzed by Cytoscape software to identify the hub genes. After that, the expression levels of hub gene in the raw sample data were utilized as the variable for principal component analysis (PCA) and the gene expression ridgeline plot. Receiver operating characteristic (ROC) curves were generated to assess the diagnostic accuracy of hub genes by calculating the area under the ROC curve (AUC).

### Animal models and treatment

Total 24 SD male rats (6-week-old, 210–220 g), obtained from SiPeiFu Animals Biotechnology (Beijing, China) were divided in to following groups: the control group, OA group, OA+LV-NC group, and OA+LV-ITGB1 group (6 rats per group). Except for the control group, rats in other group were injected with 0.2 mL of a mixed solution (a 1:1 mixture of 5% papain and 0.03 mol/L L-cysteine) into the joint cavity of the right leg on days 1, 4, and 7 to induce the OA model. The control group was injected with 0.2 mL of saline.

For the OA+LV-ITGB1 group, after the last papain and cysteine injection, ITGB1 overexpression was induced by local intra-articular injection of lentivirus (Genechem, Shanghai, China), and ITGB1 overexpressed lentivirus transfected cells (20 μL) were injected locally every 7 days for 4 weeks. At the same time, rats in OA+LV-NC group were injected with 20 μL of control lentivirus transfected cells for 4 weeks. Rats were anesthetized with 3% sodium phenobarbital (30 mg/kg) to kill after 6 days of the last papain and cysteine injection. The articular cartilage and serum of rats were collected for subsequent experiments.

All the animal operations performed in this study were followed the guideline of institutional and governmental for the ethical use of animals and approved by the Institutional Animal Care and Use Committee.

### Histological observations

The cartilage tissues (*n* = 6/group) were isolated from rats, fixed with 4% polyformaldehyde, and made into 4 μm sections after paraffin embedding. Then sections were added with Safranin O and hematoxylin & eosin (HE) sating (Sigma, St. Louis, MO, USA). Then the neutral gum seal was covered with a cover glass, and the staining results were observed under an optical microscope and photographed. OA severity was graded according to Osteoarthritis Research Society International (OARSI) scoring system to evaluate the extent of cartilage deterioration as previously described [35].

### Immunohistochemistry (IHC) analysis

In brief, the cartilage sections (*n* = 6/group) were deparaffinized, rehydrated, and antigen-retrieved in sodium citrate buffer (10 mM, pH=6.0) for 20 min, followed by treatment with 0.3% hydrogen peroxide. The sections were blocked in 3% Bovine Serum Albumin and incubated with primary antibody: anti-caspase3 (1:400; ab184787; Abcam, Cambridge, MA, USA) overnight at 4 °C. After that, the sections were incubated with horseradish peroxidase-labeled goat anti-rabbit IgG (1:200, ab288151, Abcam), followed by reacting with diaminobenzidine and counterstaining with hematoxylin.

### Cell culture and treatment

Human normal chondrocytes (C28/I2) were purchased from National Collection of Authenticated Cell Cultures (Shanghai, China). The cells were cultured in RPMI-1640 medium containing 10% FBS. After that, cells were cultured in 5 µg/mL LPS for 24 h to induce inflammation. The oe-ITGB1 (ITGB1 overexpression) plasmids and oe-NC plasmids were transfected into cells with Lipofectamine 3000 Reagent (Thermo fisher scientific, Waltham, MA, USA). The LPS+oe-ITGB1 cells were divided into two groups. cAMP pathway inhibitor, H89 (MedChemExpress, NJ, USA; 30 μΜ) was applied to explore the impact of cAMP pathway on cells.

### Enzyme-linked immunosorbent assay (ELISA)

The concentrations of TNF-α, IL-1β, IL-6 and cAMP in serum and cells were detected using commercial ELISA kits (Esebio, Shanghai, China), following the kit instructions. And the absorbance at 450 nm was detected by a microplate reader.

### Real-time quantitative PCR (RT-qPCR)

The extraction of total RNA of cartilage tissues and chondrocytes was carried out with TRIZOL reagent (Invitrogen, Carlsbad, USA). RNA was transcribed to cDNA using Double-Strand cDNA Synthesis Kits (Applied Biosystems, Foster City, CA, USA), and the reaction procedures were as follows: 25 °C for 5 min; 42 °C for 30 min; 85 °C for 5 s. The RT-qPCR experiment was performed using the ABI7500 quantitative PCR instrument (Applied Biosystems) with the following procedures: pre-denatured at 95 °C for 30 s, denatured at 95 °C for 10 s, annealed at 60 °C for 30 s, and 40 cycles. GAPDH was employed to correct and standardize the expression of the target gene and the relative expression levels of target genes were analyzed using the 2-^ΔΔCt^ method. The primer sequences were shown in Additional file 1: Table S1.

### Western blot

Proteins of cartilage tissues and chondrocytes were extracted using RIPA lysis buffer (Solarbio, Beijing, China), and the concentration was measured with the BCA protein assay kit (SWbio, Shanghai, China). The protein samples were separated by SDS-PAGE and transferred onto polyvinylidene fluoride membranes (Millipore, Danvers, USA). After blocking with 5% skim milk, the membranes were incubated with primary antibodies overnight at 4 °C. The primary antibodies used in this study were as follows: anti-ITGB1 (1:1000, ab52971, Abcam), anti-PKA (1:1000, ab216572, Abcam), anti-p-PKA (1:1000, ab75991, Abcam), and anti-β-actin (1:2000, ab8227, Abcam). Subsequently, the membranes were incubated with the secondary antibody (1:5000, ab288151, Abcam) for 1 h at room temperature. Target proteins were quantified by Image J software.

### Cell viability detection

The viability of cells was determined using a Cell Counting Kit-8 (CCK-8) kit (Beyotime, Shanghai, China). In brief, cells that seeded in 96-well plate (1 × 10^4^ cells/well) were cultured for 1 d, 2 d, and 3 d, respectively. Subsequently, cells were added with CCK-8 reagent for 2 h incubation, and the optical density at 450 nm was then detected under a microplate reader.

### Apoptosis evaluation

The apoptosis rates of chondrocytes were determined using flow cytometry analysis with an Annexin V-FITC/PI Apoptosis kit (Share Bio, Shanghai, China). For Annexin V/PI staining, cells were resuspended with Annexin V labeled FITC (5 µL) and incubated at room temperature for 5 min. Then 10 µL proprium iodide solution and 400 µL PBS were added and completely mixed. Apoptosis of cells was detected and analyzed by a flow cytometry.

### Statistical analysis

The statistical analyses in this study were performed using GraphPad Prism 7.0 software (GraphPad Software, Inc., La Jolla, CA, USA). Data were presented as mean ± standard deviation. Unpaired T tests and One-way analysis of variance (ANOVA) followed by Tukey post hoc analysis were applied to evaluate differences between two groups. *p* < 0.05 was considered to be statistically significant.

## Results

### Identification of DEGs

**Fig. 1:**
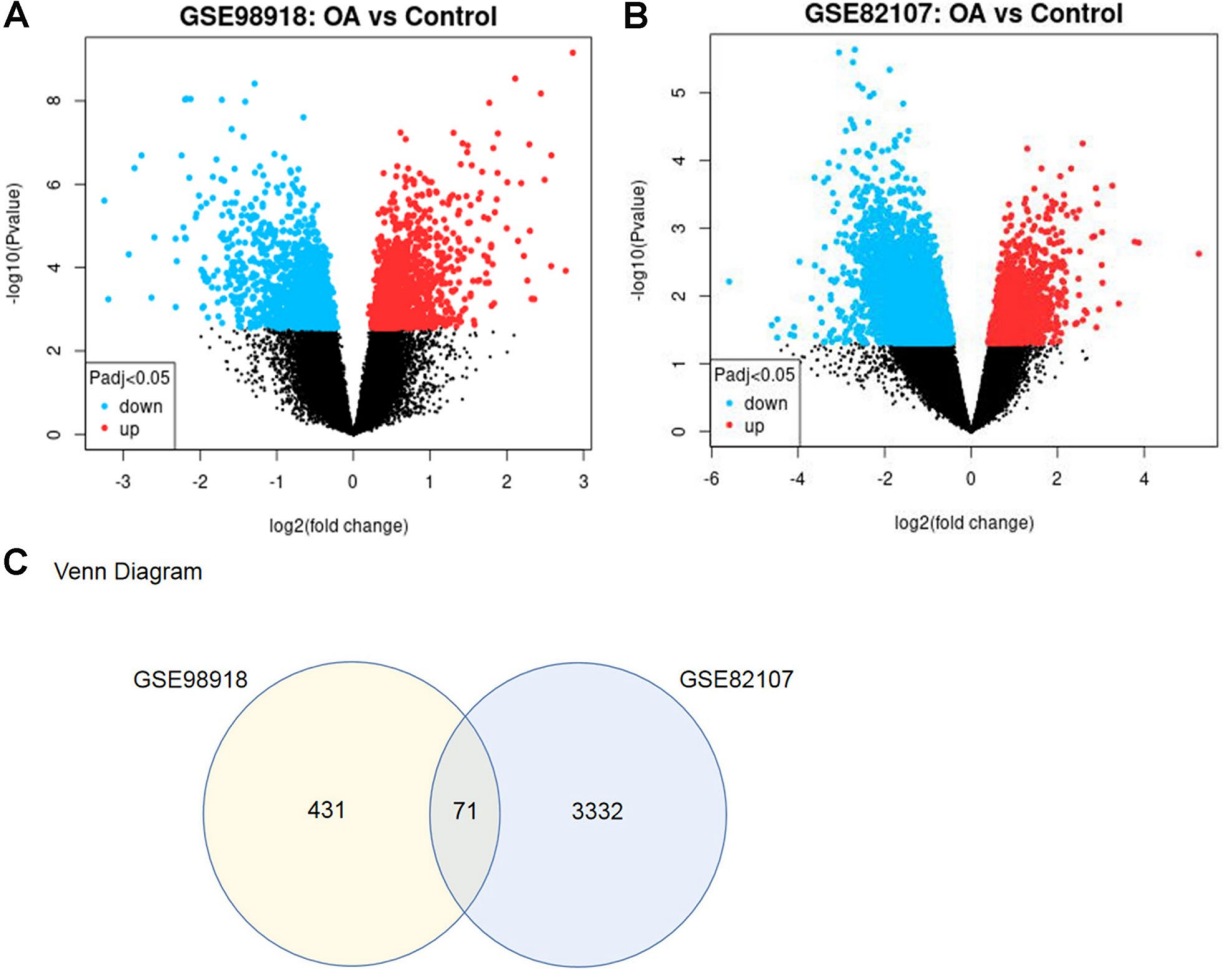
Differentially expressed genes (DEGs) identification. **A**, **B** Volcano plots of GSE98918 and GSE82107 DEGs; the abscissa is log2FoldChange, and the ordinate is -log10. Red dots represented up-regulated DEGs, blue dots represented down-regulated DEGs, gray dots represented genes which were not differentially expressed. **C** Venn diagram of DEGs. The sum of the numbers in each circle represents the number of DEGs in the dataset, and the overlapping part of the circle represents the common DEGs between GSE98918 and GSE82107

Two datasets, GSE98918 and GSE82107 were screened out, including 12 OA samples versus 12 healthy controls in GSE98918 dataset and 10 OA samples versus 7 healthy controls in GSE82107 dataset. Total 502 DEGs were identified from GSE98918, and 3403 DEGs were identified from GSE82107. The 191 up-regulated and 311 down-regulated genes in GSE98918 dataset as well as the 639 up-regulated and 2764 down-regulated genes in GSE82107 dataset were visualized by volcano plots (Fig. 1A, B). The Venn diagram showed 71 common DGEs between GSE98918 and GSE82107 (Fig. 1C). The identified samples were centered and numerically distributed up to standard, indicating that the microarray data had excellent quality and cross comparability (Additional file 1: Fig. S1A, B). In addition, the top 15 DEGs were visualized by a heatmap (Additional file 1: Fig. S1C) (Additional file 1: Table S2).

### Enrichment analysis

**Fig. 2:**
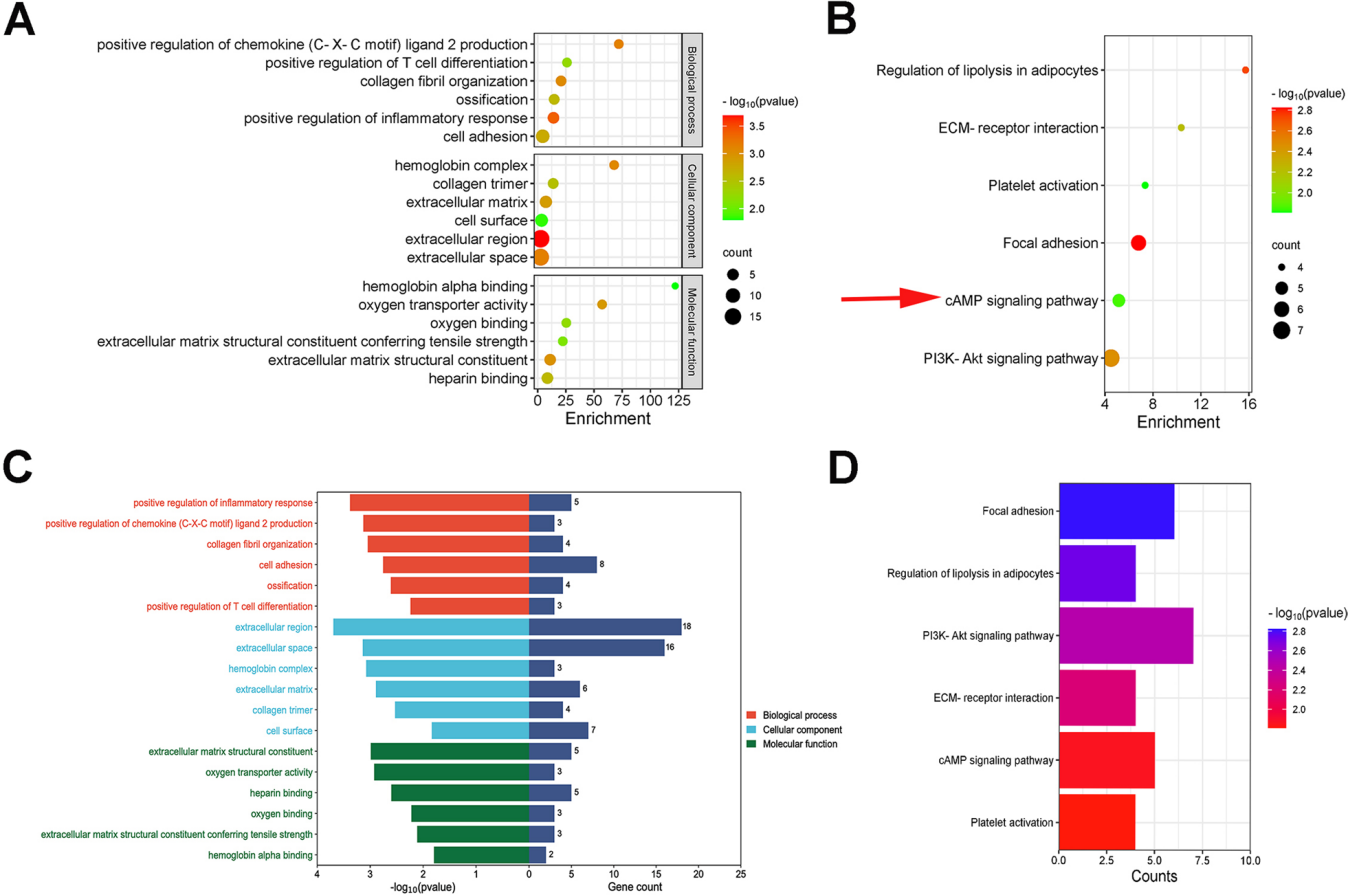
Gene Ontology (GO) and Kyoto Encyclopedia of Genes and Genomes (KEGG) analysis of common DEGs. **A**, **B** Bubble charts of GO and KEGG enrichment analysis result; the color depth of the points indicates the corrected *p*-value, and the size of the points refers to the number of genes involved. **C**, **D** Bar charts of GO and KEGG enrichment analysis result

The DEGs significantly enriched in BP were “positive regulation of chemokine (C-X-C motif) ligand 2 production,” “positive regulation of T cell differentiation,” “collagen fibril organization,” “ossification,” “positive regulation of inflammatory response”, and “cell adhesion;” the DEGs significantly enriched in MF were “hemoglobin alpha binding,” “oxygen transporter activity,” “oxygen binding,” “extracellular matrix structural constituent conferring tensile strength,” “extracellular matrix structural constituent,” and “heparin binding;” and the DEGs significantly enriched in were “hemoglobin complex,” “collagen trimer”, “extracellular matrix,” “cell surface,” “extracellular region,” and “extracellular space” (Additional file 1: Table S3). KEGG pathway analysis revealed that the pathways enriched by dysregulated DEGs included “Regulation of lipolysis in adipocytes,” “ECM-receptor interaction,” “Platelet activation,” “Focal adhesion,” “cAMP signaling pathway,” and “PI3K- Akt signaling pathway” (Additional file 1: Table S4). The most significant GO terms and KEGG pathways were shown in bubble plots visually (Fig. 2).

### PPI network construction and hub genes analysis

**Fig. 3:**
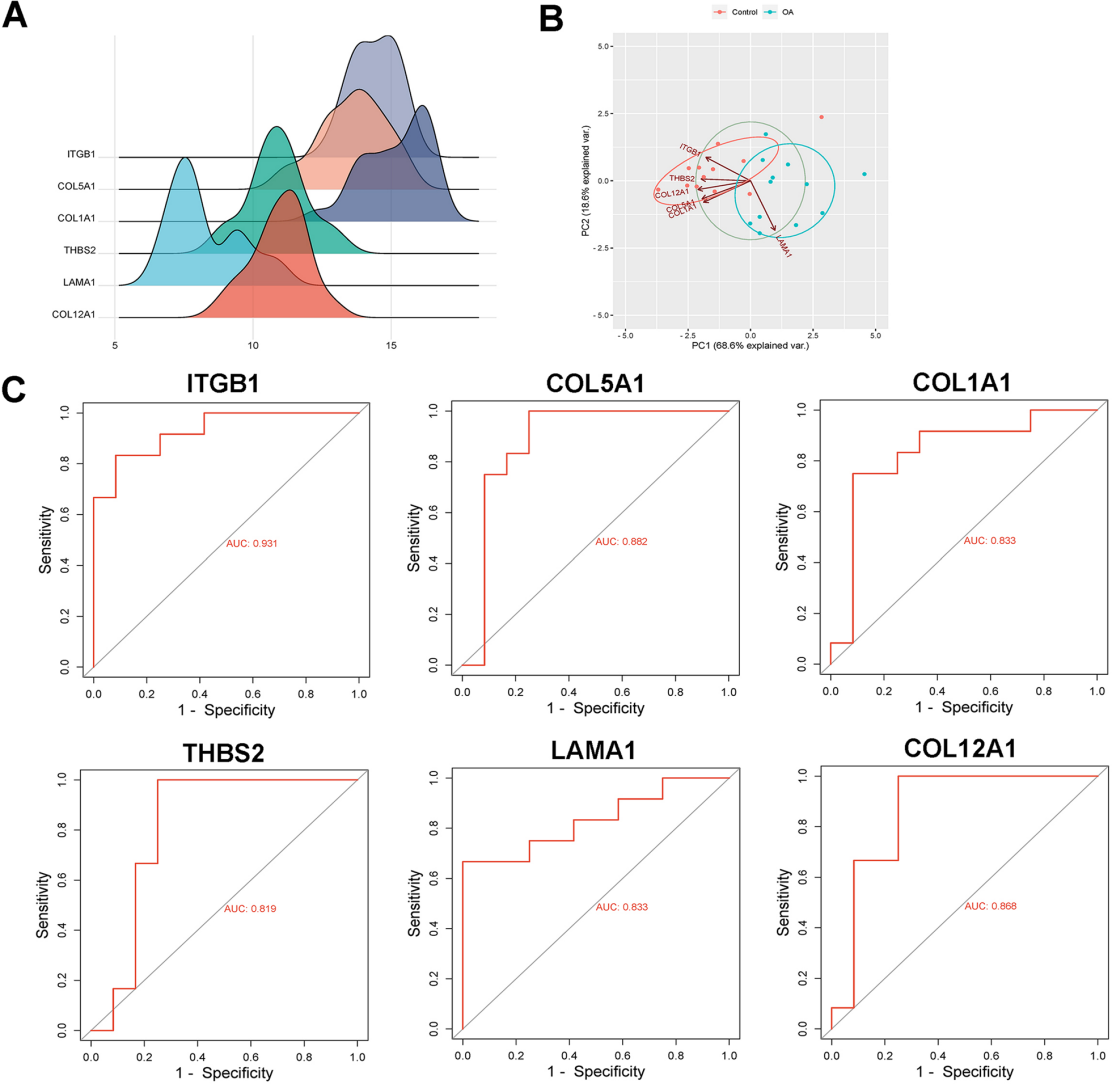
Hub genes analysis. **A** Hub genes ridgeline plot. The horizontal coordinate was the expression of hub gene, the shape of the mountain indicated the dispersion between a set of data, and the height was the number of samples corresponding to the amount of gene expression. **B** Hub genes principal component analysis diagram. The axes PC1 and PC2 in the figure were the first and second principal components; Dots represented samples, and different colors represented different groups. **C** Receiver operating characteristic curves of selected genes ITGB1, COL5A1, COL1A1, THBS2, LAMA1, and COL12A1

**Fig. 4:**
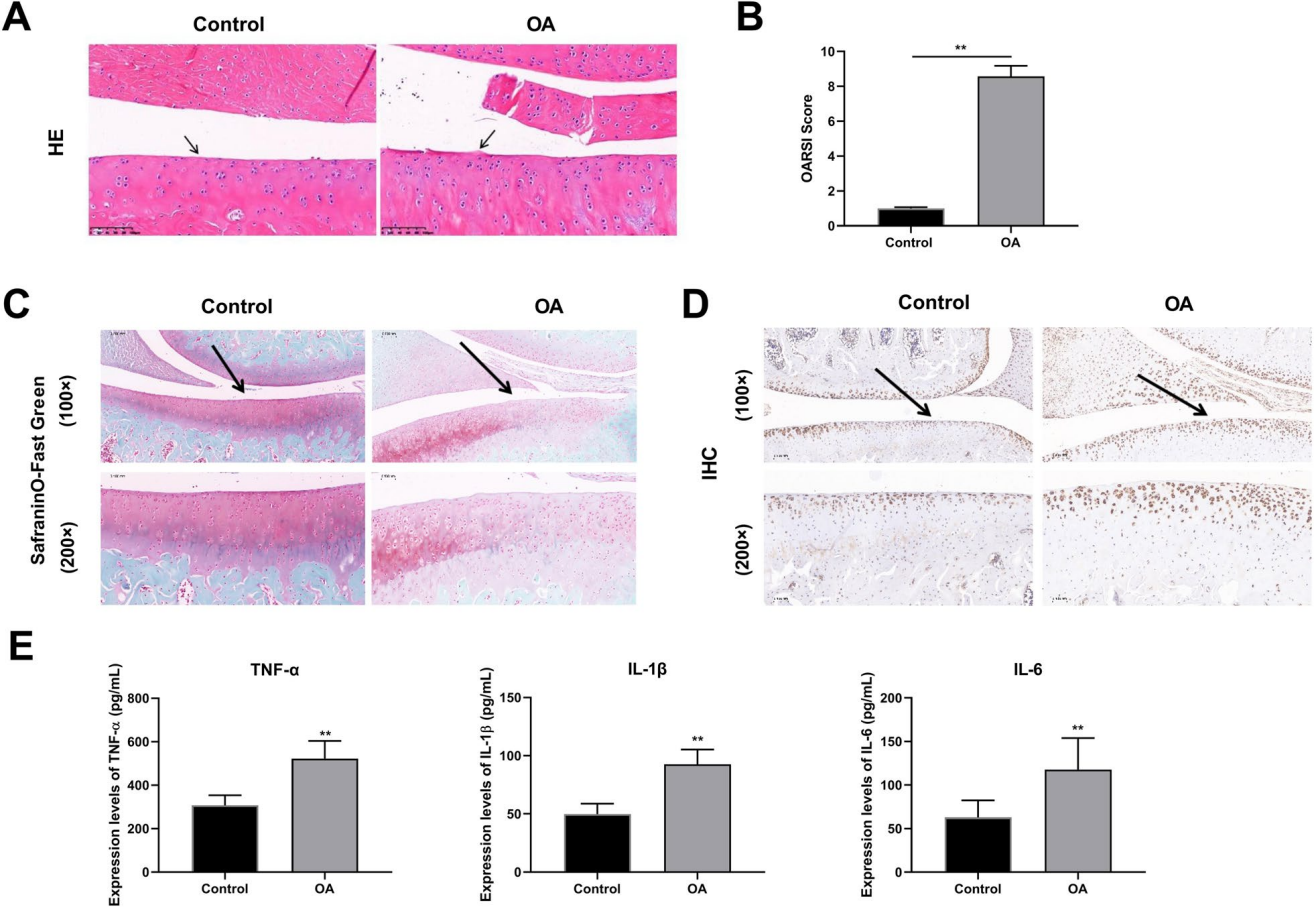
Pathological characteristics of osteoarthritis (OA) model rats (*N* = 6). **A** Representative images of hematoxylin & eosin (HE; Amplification: 200 ×, Scale: 100 µm). **B** Osteoarthritis Research Society International (OARSI) scores of articular cartilages. **C** Respresentative images of safranin O staining (Amplification: 200 ×, Scale: 100 µm). **D** Immunohistochemistry of cartilage sections from different groups (Amplification: 200 ×, Scale: 100 μm). Solid arrows show cartilage destruction. **E** Inflammatory factors levels of serum, interleukin-1β (IL-1β), tumor necrosis factor α (TNF-α), and interleukin-6 (IL-6). ^**^p < 0.01

The PPI network model was visualized using Cytoscape software (Additional file 1: Fig. S2). Among which, a key module consisting of 17 hub genes were obtained from a PPI network, including 6 nodes and 12 edges (Additional file 1: Fig. S3A). Eventually, 6 hub genes, ITGB1, COL5A1, COL1A1, THBS2, LAMA1, and COL12A1 were identified. The expression of hub genes was employed to draw the ridgeline plot (Fig. 3A). After R language processing, PC1 and PC2 were obtained, which could provide a variance explanation rate of 87.2%. Scatter plot showed that the two groups of samples were well separated, further confirming the effectiveness of PC1 and PC2 for distinguishing between control and OA group samples (Fig. 3B). To verify the diagnostic value of hub genes, ROC curves of ITGB1, COL5A1, COL1A1, THBS2, LAMA1, and COL12A1 were plotted (Fig. 3C). The ROC curve of ITGB1 revealed that the true positive rate was 93.1%, indicating that ITGB1 could be used as a discriminator of OA. In addition, enrichment chord plot revealed that hub genes were mainly enriched in “activates PTK2 signaling,” “core matrisome,” and “collagens” (1: Fig. 4). After the above hub gene analysis and literature search, we selected ITGB1 as the target gene.

### Characterization of OA rat models

HE staining revealed that the chondrocytes of the articular cartilage were disorderedly lined and tended to hypertrophic in the model group, indicating that the cartilage surface was injured. Early OA-like manifestations were also present, including chondrocytes loss, cartilage fibrillation, and erosion (Fig. 4A, B). IHC results revealed that apoptotic cells in the cartilage region were apparently more in the model group than those in the control group (Fig. 4C). In addition, the OARSI scores were improved in OA model rats compared with those in controls (Fig. 4D). And the concentrations of TNF-α, IL-1β, and IL-6 were significantly evaluated in the OA group, compared to control group (Fig. 4E). The aforementioned findings showed that the OA rat model was successfully established.

**Fig. 5:**
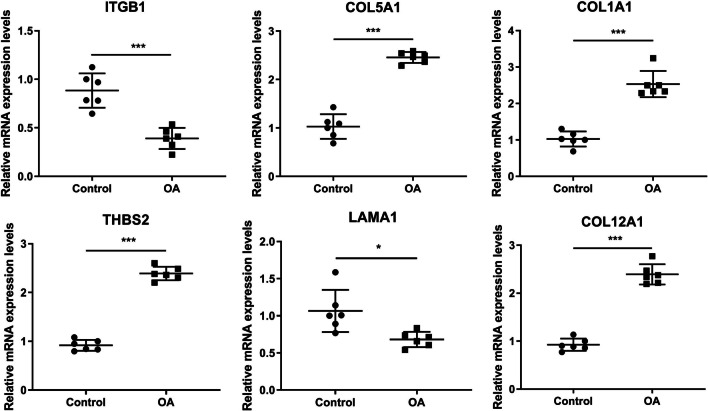
The mRNA expression levels of ITGB1, COL5A1, COL1A1, THBS2, LAMA1, and COL12A1 in tissues were detected by real-time quantitative PCR (RT-qPCR). (*N* = 6), ^***^p < 0.001

RT-qPCR results showed that the mRNA expression levels of ITGB1, COL5A1, THBS2, and COL12A1 were significantly down-regulated, while the mRNA expression levels of COL1A1 and LAMA1 were up-regulated in OA, which consisted with the results of bioinformatics analysis, further confirming the reliability of bioinformatics analysis (Fig. 5).

### Overexpression of ITGB1 inhibits the development of OA in rat

**Fig. 6:**
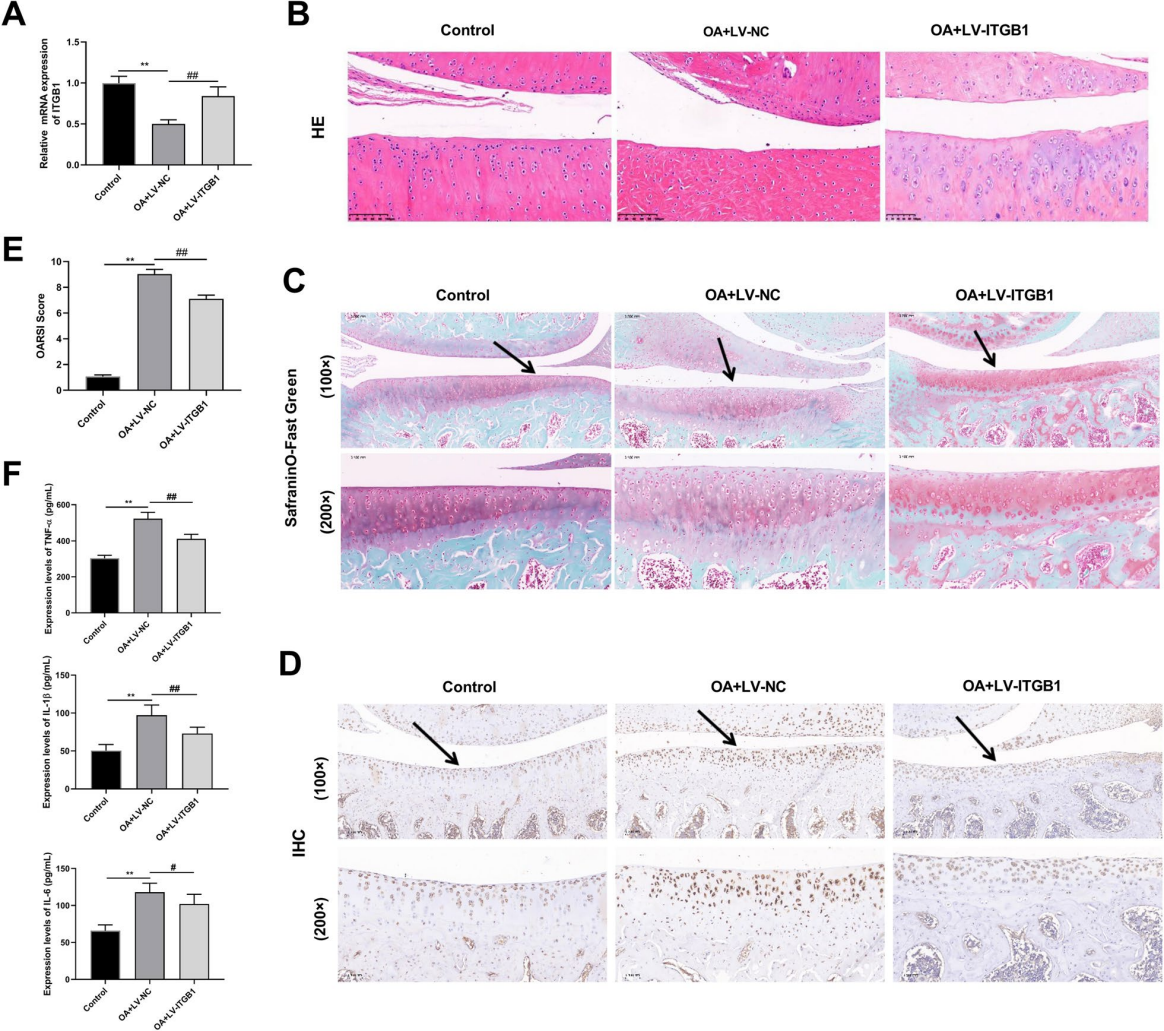
ITGB1 improves OA symptoms in rats (*N* = 3). **A** The mRNA expression level of ITGB1 was detected by RT-qPCR. **B–D** Results of H&E, safranin O staining and IHC of rat cartilage tissue (Amplification: 200 × , Scale: 100 μm). **E** OARSI scores of articular cartilage. **F** Enzyme-linked immunosorbent assay (ELISA) was used to detect the levels of IL-1β, TNF-α and IL-6. ^**^p < 0.01 versus Control; ^##^*p* < 0.01, ^#^*p* < 0.05 versus OA+LV-NC

RT-qPCR showed that ITGB1 expression was obviously up-regulated in the OA+LV-ITGB1 group (Fig. 6A). More normal cells and orderly arrangement of cells in cartilage tissue of OA+LV-ITGB1 group were observed and the level of cartilage surface wear and apoptotic cells, as well as the OARSI score were reduced, compare with the OA+LV-NC group, suggesting that the overexpression of ITGB1 improved the symptoms of OA in rats (Fig. 6B–E). ELISA results showed that the overexpression of ITGB1 decreased the concentrations of inflammatory factors (TNF-α, IL-1β, and IL-6) in the serum of OA rats (Fig. 6F). ITGB1 overexpression attenuated inflammatory injury and inhibited OA progression in OA model rats.

### ITGB1 alleviates LPS-induced chondrocyte damage via activating the cAMP signaling pathway

**Fig. 7:**
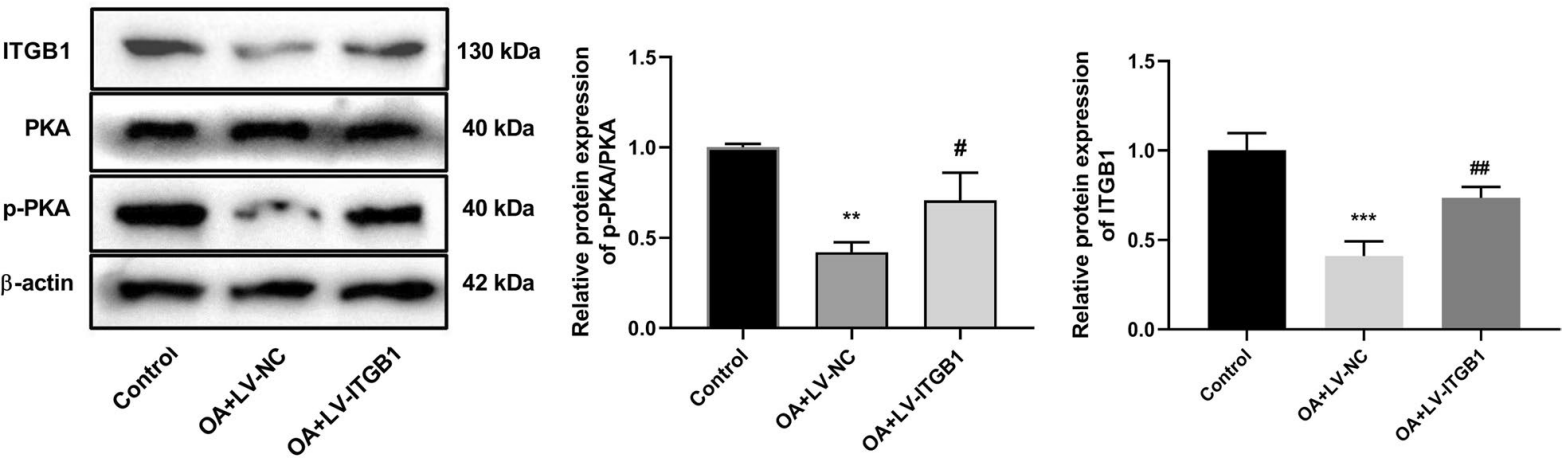
ITGB1 promotes the activation of cAMP signaling pathway in rats (*N* = 3). The expression levels of ITGB1, PKA, and p-PKA were detected by western blot. ^**^p < 0.01, ^***^p < 0.001 versus Control; ^#^*p* < 0.05, ^##^*p* < 0.01 versus OA+LV-NC

Western blot results suggested that the expression level of ITGB1 in OA rats of OA+LV-NC group was lower than that in control group. And the expression level of ITGB1 in OA+LV-ITGB1 group was significantly increased, compared to the OA+LV-NC group. After ITGB1 overexpression, the expression levels of p-PKA/PKA were significantly increased, compared with rats in the OA+LV-NC group (Fig. 7). Thus, it implied that ITGB1 activated the cAMP signaling pathway in the OA model.

**Fig. 8:**
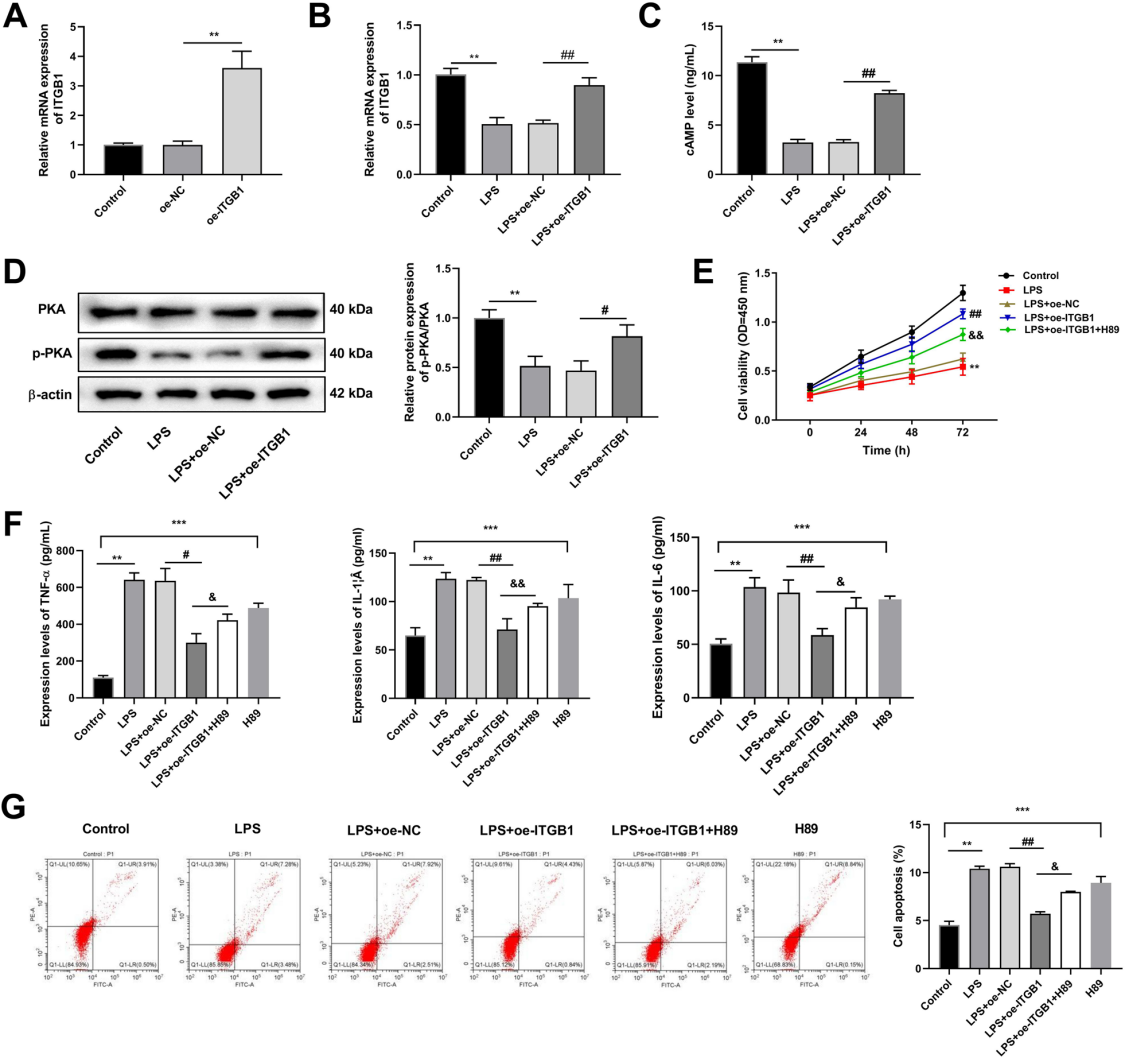
ITGB1 ameliorates chondrocyte damage through the cAMP pathway in C28/I2 cells (*N* = 3). **A** RT-qPCR was used to detect ITGB1 expression in normal chondrocytes of each group. **B** RT-qPCR was used to detect ITGB1 expression in OA model chondrocytes of each group. **C** ELISA detected the cAMP concentration in each group. **D** The levels of PKA and p-PKA were detected by western blot. **E** Cell Counting Kit-8 assay detected cell viability. **F** The levels of IL-1β, TNF-α, and IL-6 were detected by ELISA. **G** Apoptotic chondrocytes were quantified by flow cytometry analysis with fluorescence staining. ^**^p < 0.01, ^***^p < 0.001 versus Control; ^#^*p* < 0.05, ^##^*p* < 0.01 versus LPS+oe-NC; ^&^*p* < 0.05, ^&&^*p* < 0.01 versus LPS+oe-ITGB1

RT-qPCR results demonstrated that ITGB1 expression was considerably higher in the oe-ITGB1 group than that in the oe-NC group (Fig. 8A). Compared with the control group, the mRNA expression level of ITGB1 decreased after LPS treatment, while the expression level of ITGB1 in LPS+oe-ITGB1 group was significantly higher, compared with LPS+oe-NC group (Fig. 8B).

Overexpression of ITGB1 significantly increased cAMP concentration in LPS-induced C28/I2 cells, compared to LPS+oe-NC (Fig. 8C). In addition, western blot results illustrated that overexpression of ITGB1 evaluated the expression level of p-PKA protein (Fig. 8D).

CCK-8 assay revealed that ITGB1 overexpression promoted the viability, compared to the LPS group (Fig. 8E). In addition, compared with the control group, the pathway inhibitor, H89 significantly increased the expression of TNF-α, IL-1β, and IL-6 in C28/I2 cells. Conversely, ITGB1 overexpression decreased the expression of TNF-α, IL-1β, and IL-6 in LPS-induced C28/I2 cells, while the treatment of pathway inhibitors reversed the positive effects of ITGB1 on LPS-induced C28/I2 cells (Fig. 8F). For the apoptosis, the H89 treatment considerably promoted the apoptosis of C28/I2 cells, compared to the control group, while the apoptosis rate was significantly lower in LPS+oe-ITGB1 group, compared to the LPS+oe-NC group. Moreover, the treatment of pathway inhibitors caused an increase in apoptosis rate in LPS+oe-ITGB1+H89 group, compared to LPS+oe-ITGB1+H89 group (Fig. 8G).

## Discussion

Over-activation of the inflammatory response plays a central role in occurrence and development of OA [36–38]. Additionally, chondrocyte apoptosis is also a crucial index contributing to the progression of OA [39, 40]. Herein, we searched for the intrinsic molecular mechanisms that inhibit chondrocyte inflammation and apoptosis. In present study, we identified ITGB1, COL5A1, COL1A1, THBS2, LAMA1, and COL12A1 as the hub genes of OA with good prediction value. And ITGB1 was selected as key gene of our study. ITGB1 was down-regulated in the OA rat model. Furthermore, overexpression of ITGB1 ameliorated the pathological injury and reduced the apoptotic cells, as well as inflammatory factor levels in cartilage tissue of OA rat. ITGB1 promoted protein expression of cAMP pathway in vitro and in vivo. Additionally, in vitro model, we found that overexpression of ITGB1 promoted the proliferation while inhibited apoptosis and inflammatory response via stimulating cAMP pathway.

ITGB1, COL5A1, COL1A1, THBS2, LAMA1, and COL12A1 have been widely reported in OA and other inflammatory diseases. Based on GSE20907, GSE45006, and GSE45550 datasets, ITGB1 is screened out as hub genes of spinal cord injury after weighted correlation network analysis [41]. After analyzing the mRNA, miRNA, and DNA methylation expression profiles of healthy or osteoarthritic articular cartilage samples, COL5A1 is identified as one of the hub genes that play an important role in the occurrence and development of OA [42]. COL1A1 is identified as key gene in the knee cartilage tissue of healthy controls and patients with OA based on the GSE169077 gene chip dataset [43]. Through proteomic analysis of chondrocytes, COL12A1 is found to be a key gene in acupotomy intervention for the treatment of OA [44]. Based on the gene expression profiles of GSE51588 and GSE114007, and the gene methylation chip data GSE63695, THBS2 is determined as a possible biomarker in end-stage of OA [45]. mRNA analysis reveals that the expression level of LAMA1 in peripheral blood mononuclear cells of OA patients is significantly lower than that of normal control group, showing a statistically significant correlation with OA [46]. In our research, after analyzing GSE98918 and GSE82107 datasets, ITGB1, COL5A1, COL1A1, THBS2, LAMA1, and COL12A1 were screened out as hub genes of OA with high prediction value, which might be important diagnostic markers for OA.

ITGB1 is a crucial mediator of the treatment of OA. Tropoelastin promotes the properties of infrapatellar adipose pad mesenchymal stem cells and protects knee cartilage and inhibits the development of OA by enhancing cell adhesion and activating ITGB1 [47]. The activation of ITGB1-induced ERK1/2 phosphorylation can inhibit chondrocyte hypertrophy, thus suppressing the progression of OA in bone [48]. Additionally, ITGB1 is involved in regulating various inflammatory diseases. In gouty arthritis with cartilage damage, inflammation is enhanced by activating the ITGB1-dependent TLR2/4-NF-κB signaling pathway [49]. Extracellular vesicle-derived circular ITGB1 can exacerbate heart damage and inflammation, which can cause acute myocardial infarction [50]. In ITGB1-kickout mice, genetic deletion of monocyte chemoattractant protein-1 receptor impairs the recruitment of monocyte-derived macrophages, which can accelerate the progression of inflammation and severely pre-matures the destruction of emphysema [51]. We found that ITGB1 was down-regulated in OA progression and ITGB1 overexpression decreased the concentrations of inflammatory factor and ameliorated the pathological injury. In vitro, overexpression of ITGB1 promoted the proliferation, while inhibited apoptosis. All in all, overexpression of ITGB1 inhibits the development of OA.

In our study, overexpression of ITGB1 activated cAMP pathway in OA rats and cAMP inhibitor, H89 reversed the inhibitory effect of ITGB1 on OA development. cAMP is a novel regulator of anti-inflammation, and the cAMP-dependent pathway has been widely applied for the treatment of inflammatory diseases [34]. In general, the increased concentration of cAMP mediates anti-inflammatory effects [52]. Previous studies have reported that elevated cAMP level promotes anti-inflammatory responses and inhibits cartilage degradation in patients with OA [53, 54]. Forskolin prevents OA chondrocyte mitochondrial dysfunction and attenuates synovial fibrosis by increasing cAMP levels and serves as a treatment for OA [55, 56]. Furthermore, increase in cAMP in synovial cells can alleviate synovial fibrosis by increasing production of hyaluronic acid and proteoglycan 4, which may represent a new option for treating synovial fibrosis in OA [55]. Additionally, cAMP has been reported to prevent chondrocyte mitochondrial dysfunction induced by IL-1β, which can cause a blunted response of OA chondrocytes to inflammatory stimuli [56, 57]. Taken together, ITGB1 alleviates OA by inhibiting cartilage inflammation and apoptosis via activating cAMP pathway.

In summary, we identified 6 hub genes, ITGB1, COL5A1, COL1A1, THBS2, LAMA1, and COL12A1 with diagnostic value for OA. Among them, ITGB1 is a novel gene signature with high value for OA diagnosis and treatment, which exerts a significant inhibitory effect on progression of OA via activating AMP pathway. Taken together, our findings provided a fresh understanding of the pathophysiology of OA development, which can help us to better grasp the probable mechanisms of the development of OA. It is envisaged that this paper may offer preliminary evidence for more effective therapy targets of OA.

### Supplementary Information

**Additional file 1: Fig. S1.** DEGs analysis. **A**, **B** Cross comparability evaluation of microarray data. **C** Heat map of the DEGs; red represented highly expressed genes, and blue represented low expressed genes. **Fig. S2.** Panorama of protein–protein interaction (PPI) network for DEGs. The PPI network constructed via STRING database representing the degree of gene interaction. **Fig. S3.** Identification of hub genes using Cytoscape and MCODE plugin. The PPI network constructed via STRING database representing the degree of gene interaction and hub gene modules. **A** Hub gene identified based on MCODE analysis. The lines between nodes represented interactions between genes. **B** The top 10 genes scored based on MCC method, and the color depth of genes represented the score. **Fig. S4.** Hub genes analysis. **A** The GO chord plot plotted using R package. The data consist of 3 parts: genes; logFC, for sequencing and gene block color; and GO term. **B** Matrix correlation analysis to demonstrate the correlation of genes between matrices. **Table S1.** The sequence of primers. **Table S2.** The top 15 differentially expressed genes. **Table S3.** The Top6 Go term. **Table S4.** Top6 KEGG pathway.

## Data Availability

The datasets used and/or analyzed during the current study are available from the corresponding author on reasonable request.
