# Supplementary material for: ITGB1 alleviates osteoarthritis by inhibiting cartilage inflammation and apoptosis via activating cAMP pathway
Source: J Orthop Surg Res. 2023 Nov 8;18:849. doi: 10.1186/s13018-023-04342-y (PMC10634155; doi:10.1186/s13018-023-04342-y)
Supplement: Supplementary file 1 — Additional file 1: Fig. S1. DEGs analysis. A, B Cross comparability evaluation of microarray data. C Heat map of the DEGs; red represented highly expressed genes, and blue represented low expressed genes. Fig. S2. Panorama of protein–protein interaction (PPI) network for DEGs. The PPI network constructed via STRING database representing the degree of gene interaction. Fig. S3. Identification of hub genes using Cytoscape and MCODE plugin. The PPI network constructed via STRING database representing the degree of gene interaction and hub gene modules. A Hub gene identified based on MCODE analysis. The lines between nodes represented interactions between genes. B The top 10 genes scored based on MCC method, and the color depth of genes represented the score. Fig. S4. Hub genes analysis. A The GO chord plot plotted using R package. The data consist of 3 parts: genes; logFC, for sequencing and gene block color; and GO term. B Matrix correlation analysis to demonstrate the correlation of genes between matrices. Table S1. The sequence of primers. Table S2. The top 15 differentially expressed genes. Table S3. The Top6 Go term. Table S4. Top6 KEGG pathway. [file 13018_2023_4342_MOESM1_ESM.docx]

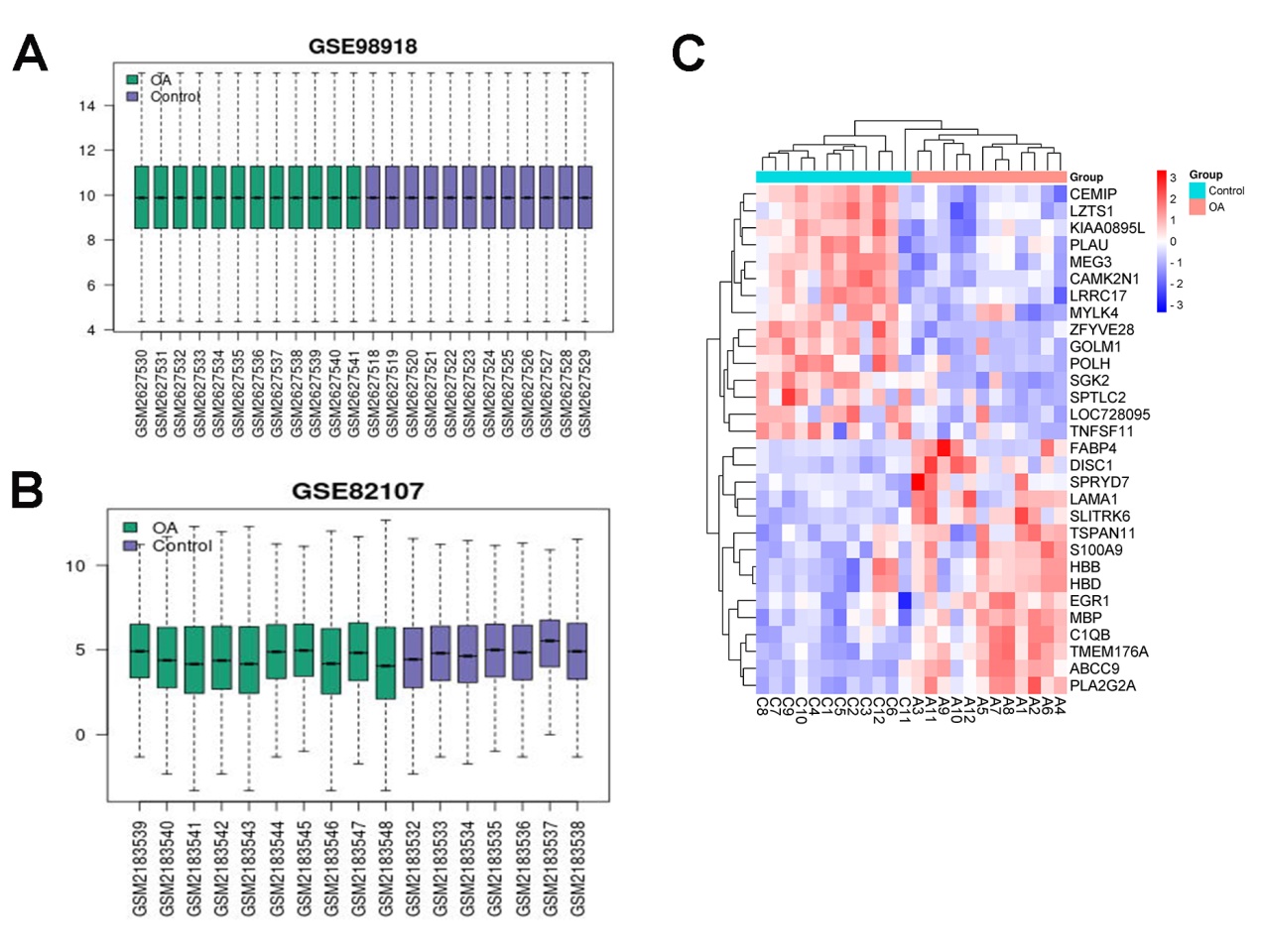


Supplement figure 1. DEGs analysis. A-B: Cross comparability evaluation of microarray data. C. Heat map of the DEGs; red represented highly expressed genes and blue represented low expressed genes.


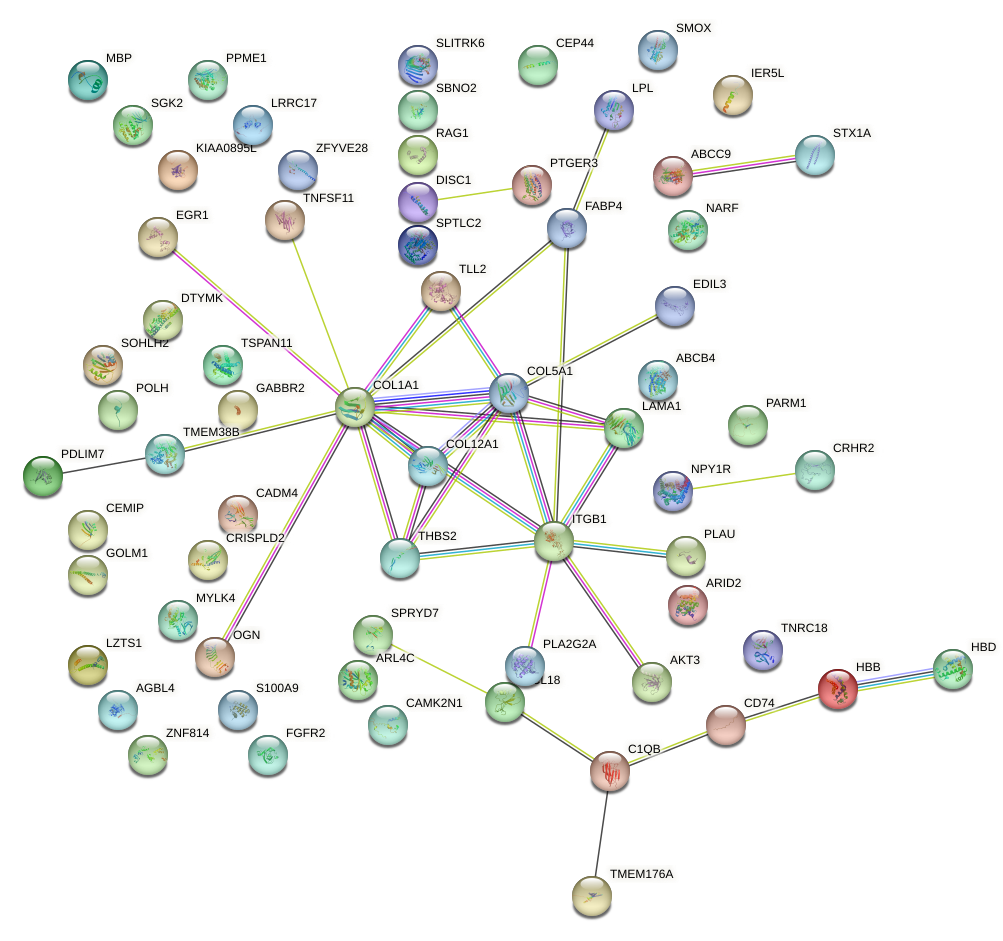


Supplement figure 2. Panorama of protein–protein interaction (PPI) network for DEGs. The PPI network constructed via STRING database representing the degree of gene interaction.


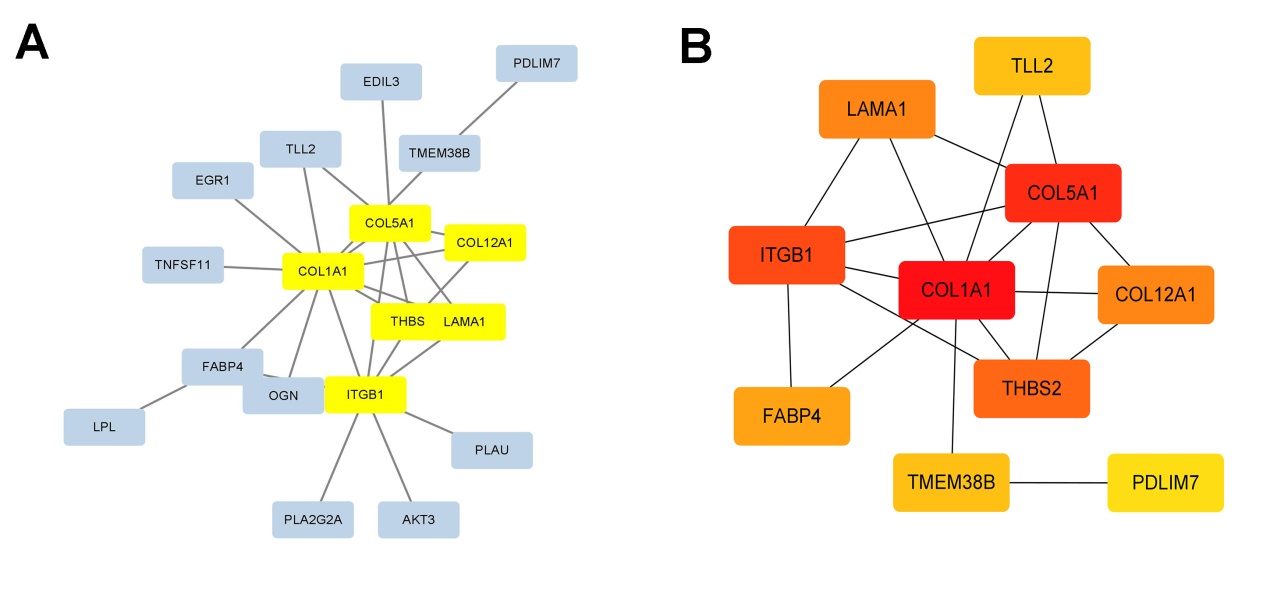


Supplement figure 3. Identification of hub genes using Cytoscape and MCODE plugin. The PPI network constructed via STRING database representing the degree of gene interaction and hub gene modules. A. Hub gene identified based on MCODE analysis. The lines between nodes represented interactions between genes. B. The top 10 genes scored based on MCC method, and the color depth of genes represented the score.


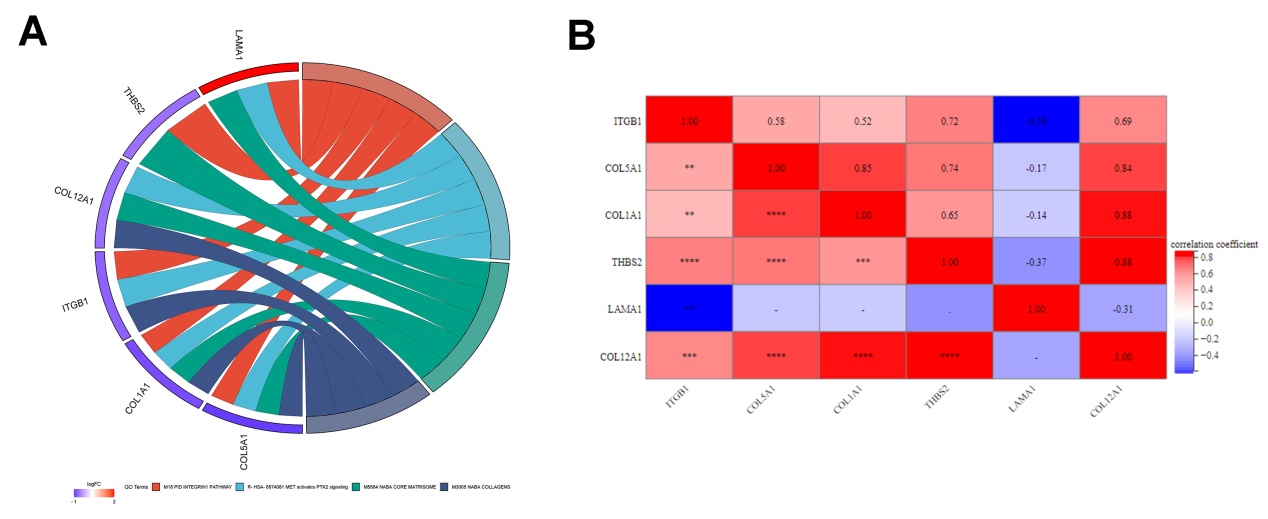


Supplement figure 4. Hub genes analysis. A. The chord plot using the GOplot R package. The data consists of 3 parts: genes; logFC, for sequencing and gene block color; and GO term. B. Matrix correlation analysis to demonstrate the correlation of genes between matrices.

**Supplement Table 1. The sequence of primers**

| Primers | Sequence (5’-3’) |
| --- | --- |
| GAPDH-F | CTCATGACCACAGTCCATGC |
| GAPDH-R | TTCAGCTCTGGGATGACCTT |
| ITGB1-F | GCCAGTGTCACCTGGAAAAT |
| ITGB1-R | TGTGCCCACTGCTGACTTAG |
| LAMA1-F | GCTCCTGCAAGGAGAATGTC |
| LAMA1-R | TGTGAAACCACATGCAGGAT |
| COL1A1-F | TGACTGGAAGAGCGGAGAGT |
| COL1A1-R | GAATCCATCGGTCATGCTCT |
| COL5A1-F | GTGGCACAGAATTGCTCTCA |
| COL5A1-F | GTGGCACAGAATTGCTCTCA |
| THBS2-F | TCGTGCGCTTTGACTACATC |
| THBS2-R | GTGCCGTCAATCCAGTAGGT |
| COL12A1-F | GGAGACAGAGGCTTCACTGG |
| COL12A1-R | ACTGCTCGCATCATGTTCTG |

**Supplement Table 2. The top 15 differentially expressed genes**

| Genes | Description | log2FoldChange | pval | up/down |
| --- | --- | --- | --- | --- |
| ABCC9 | ATP-binding cassette, sub-family C (CFTR/MRP), member 9 | 2.58 | 2.04E-07 | up |
| HBB | Hemoglobin, beta | 2.00 | 1.07E-02 | up |
| PLA2G2A | Phospholipase A2, group IIA (platelets, synovial fluid) | 1.88 | 5.36E-07 | up |
| S100A9 | S100 calcium binding protein A9 | 1.77 | 2.29E-04 | up |
| LAMA1 | Laminin, alpha 1 | 1.64 | 2.01E-04 | up |
| HBD | Hemoglobin, delta | 1.60 | 4.31E-03 | up |
| C1QB | Complement component 1, q subcomponent, B chain | 1.53 | 3.19E-06 | up |
| SPRYD7 | SPRY domain containing 7 | 1.45 | 8.70E-03 | up |
| TMEM176A | Transmembrane protein 176A | 1.42 | 1.05E-07 | up |
| EGR1 | Early growth response 1 | 1.42 | 1.10E-03 | up |
| SLITRK6 | SLIT and NTRK-like family, member 6 | 1.38 | 2.40E-05 | up |
| DISC1 | Disrupted in schizophrenia 1 | 1.38 | 2.28E-04 | up |
| TSPAN11 | Tetraspanin 11 | 1.33 | 1.53E-02 | up |
| FABP4 | Fatty acid binding protein 4, adipocyte | 1.31 | 9.55E-03 | up |
| MBP | Myelin basic protein | 1.26 | 2.34E-04 | up |
| GOLM1 | Golgi membrane protein 1 | -1.45 | 8.59E-06 | down |
| POLH | Polymerase (DNA directed), eta | -1.46 | 4.89E-04 | down |
| KIAA0895L | KIAA0895-like | -1.47 | 4.51E-05 | down |
| MYLK4 | Myosin light chain kinase family, member 4 | -1.51 | 1.04E-03 | down |
| SPTLC2 | Serine palmitoyltransferase, long chain base subunit 2 | -1.51 | 1.45E-03 | down |
| LRRC17 | Leucine rich repeat containing 17 | -1.53 | 3.34E-04 | down |
| TNFSF11 | Tumor necrosis factor (ligand) superfamily, member 11 | -1.56 | 3.34E-03 | down |
| CAMK2N1 | Calcium/calmodulin-dependent protein kinase II inhibitor 1 | -1.58 | 7.56E-05 | down |
| ZFYVE28 | Zinc finger, FYVE domain containing 28 | -1.59 | 4.74E-08 | down |
| LZTS1 | Leucine zipper, putative tumor suppressor 1 | -1.59 | 1.13E-04 | down |
| LOC728095 | uncharacterized LOC728095 | -1.85 | 2.93E-03 | down |
| PLAU | Plasminogen activator, urokinase | -1.97 | 9.31E-04 | down |
| MEG3 | Maternally expressed 3 (non-protein coding) | -1.99 | 3.52E-06 | down |
| SGK2 | Serum/glucocorticoid regulated kinase 2 | -2.06 | 6.26E-06 | down |
| CEMIP | Cell migration inducing protein, hyaluronan binding | -2.32 | 2.03E-05 | down |

**Supplement Table 3. The Top6 Go term**

| GO TERM | Term | Pvalue | Fold enrichment | Count | GeneRatio | Genes |
| --- | --- | --- | --- | --- | --- | --- |
| GOTERM-BP-DIRECT | GO:0050729 positive regulation of inflammatory response | 4.17E-04 | 14.05 | 5 | 7.14 | FABP4, CAMK2N1, PLA2G2A, LPL, S100A9 |
| GOTERM-BP-DIRECT | GO:2000343 positive regulation of chemokine (C-X-C motif) ligand 2 production | 7.47E-04 | 71.96 | 3 | 4.29 | CD74, LPL, MBP |
| GOTERM-BP-DIRECT | GO:0030199 collagen fibril organization | 9.00E-04 | 20.79 | 4 | 5.71 | COL1A1, COL5A1, COL12A1, TLL2 |
| GOTERM-BP-DIRECT | GO:0007155 cell adhesion | 1.76E-03 | 4.49 | 8 | 11.43 | ITGB1, COL1A1, CADM4, COL5A1, LAMA1, COL12A1, THBS2, EDIL3 |
| GOTERM-BP-DIRECT | GO:0001503 ossification | 2.46E-03 | 14.67 | 4 | 5.71 | COL1A1, LRRC17, TNFSF11, PDLIM7 |
| GOTERM-BP-DIRECT | GO:0045582 positive regulation of T cell differentiation | 5.76E-03 | 25.99 | 3 | 4.29 | CD74, ARID2, RAG1 |
| GOTERM-CC-DIRECT | GO:0005576 extracellular region | 2.02E-04 | 2.67 | 18 | 25.71 | C1QB, CEMIP, LAMA1, PLA2G2A, COL12A1, HBB, LPL, THBS2, COL1A1, COL5A1, CRISPLD2, PLAU, OGN, TLL2, TNFSF11, S100A9, STX1A, FGFR2 |
| GOTERM-CC-DIRECT | GO:0005615 extracellular space | 7.24E-04 | 2.61 | 16 | 22.86 | GOLM1, LAMA1, PLA2G2A, COL12A1, LRRC17, HBB, LPL, COL1A1, COL5A1, CRISPLD2, PLAU, OGN, TLL2, TNFSF11, CCL18, S100A9 |
| GOTERM-CC-DIRECT | GO:0005833 hemoglobin complex | 8.46E-04 | 67.85 | 3 | 4.29 | HBB, HBD |
| GOTERM-CC-DIRECT | GO:0031012 extracellular matrix | 1.29E-03 | 7.31 | 6 | 8.57 | COL1A1, COL5A1, CRISPLD2, LAMA1, OGN, LRRC17 |
| GOTERM-CC-DIRECT | GO:0005581 collagen trimer | 2.96E-03 | 13.77 | 4 | 5.71 | C1QB, COL1A1, COL5A1, COL12A1 |
| GOTERM-CC-DIRECT | GO:0009986 cell surface | 1.46E-02 | 3.45 | 7 | 10 | ITGB1, CD74, PLAU, SLITRK6, LPL, MBP, FGFR2 |
| GOTERM-MF-DIRECT | GO:0005201 extracellular matrix structural constituent | 1.03E-03 | 11.05 | 5 | 7.14 | COL1A1, COL5A1, LAMA1, THBS2, EDIL3 |
| GOTERM-MF-DIRECT | GO:0005344 oxygen transporter activity | 1.19E-03 | 57.18 | 3 | 4.29 | HBB, HBD |
| GOTERM-MF-DIRECT | GO:0008201 heparin binding | 2.50E-03 | 8.66 | 5 | 7.14 | COL5A1, CRISPLD2, LPL, THBS2, FGFR2 |
| GOTERM-MF-DIRECT | GO:0019825 oxygen binding | 6.01E-03 | 25.41 | 3 | 4.29 | HBB, HBD |
| GOTERM-MF-DIRECT | GO:0030020 extracellular matrix structural constituent conferring tensile strength | 7.74E-03 | 22.31 | 3 | 4.29 | COL1A1, COL5A1, COL12A1 |
| GOTERM-MF-DIRECT | GO:0031721 hemoglobin alpha binding | 1.60E-02 | 121.99 | 2 | 2.86 | HBB, HBD |

**Supplement Table 4. Top6 KEGG pathway**

| KEGG-PATHWAY | Term | Pvalue | Fold enrichment | Count | GeneRatio |
| --- | --- | --- | --- | --- | --- |
| KEGG-PATHWAY | hsa04510: Focal adhesion | 1.50E-03 | 6.8 | 6 | 8.57 |
| KEGG-PATHWAY | hsa04923: Regulation of lipolysis in adipocytes | 1.87E-03 | 15.72 | 4 | 5.71 |
| KEGG-PATHWAY | hsa04151: PI3K-Akt signaling pathway | 3.47E-03 | 4.51 | 7 | 10 |
| KEGG-PATHWAY | hsa04512: ECM-receptor interaction | 6.09E-03 | 10.36 | 4 | 5.71 |
| KEGG-PATHWAY | hsa04024: cAMP signaling pathway | 1.39E-02 | 5.16 | 5 | 7.14 |
| KEGG-PATHWAY | hsa04611: Platelet activation | 1.55E-02 | 7.35 | 4 | 5.71 |
